# An fMRI investigation of posttraumatic flashbacks

**DOI:** 10.1016/j.bandc.2012.10.002

**Published:** 2013-02

**Authors:** Matthew G. Whalley, Marijn C.W. Kroes, Zoe Huntley, Michael D. Rugg, Simon W. Davis, Chris R. Brewin

**Affiliations:** a Clinical, Educational & Health Psychology, University College London, UK; b Donders Institute for Brain, Cognition and Behaviour, Nijmegen, The Netherlands; c Center for Vital Longevity, Functional Neuroimaging of Memory Laboratory, The University of Texas at Dallas, TX, USA; d Department of Experimental Psychology, University of Cambridge, UK; e Berkshire Traumatic Stress Service & Clinical Health Psychology Service, Reading, UK

**Keywords:** PTSD, Flashbacks, Memory, Dual-process, Familiarity

## Abstract

Flashbacks are a defining feature of posttraumatic stress disorder (PTSD), but there have been few studies of their neural basis. We tested predictions from a dual representation model of PTSD that, compared with ordinary episodic memories of the same traumatic event, flashbacks would be associated with activity in dorsal visual stream and related areas rather than in the medial temporal lobe. Participants with PTSD, with depression but not PTSD, and healthy controls were scanned during a recognition task with personally relevant stimuli. The contrast of flashbacks versus ordinary episodic trauma memories in PTSD was associated with increased activation in sensory and motor areas including the insula, precentral gyrus, supplementary motor area, and mid-occipital cortex. The same contrast was associated with decreased activation in the midbrain, parahippocampal gyrus, and precuneus/posterior cingulate cortex. The results were discussed in terms of theories of PTSD and dual-process models of recognition.

## Highlights

► Flashbacks in PTSD are associated with decreased rather than increased MTL activation. ► Flashbacks in PTSD are associated with activation in the insula and in motor and sensory areas. ► Flashbacks in PTSD may correspond to familiarity rather than recollection responses.

## 1 Introduction

Post-traumatic stress disorder (PTSD) has frequently been characterized as a disorder of memory (Brewin, 2011; van der Kolk, 1996). PTSD patients typically experience flashbacks, involuntary sensory images of the traumatic scene. These images are vivid, detailed, and lack temporal context, being re-experienced as though they were happening in the present (Whalley, Farmer, & Brewin, 2007). Flashbacks co-exist with ordinary episodic memories of the trauma that can be deliberately retrieved and communicated. The dual representation theory of PTSD (Brewin, 2001; Brewin, Dalgleish, & Joseph, 1996) makes an unusual prediction that, despite flashbacks being extremely vivid, they should involve less rather than more medial temporal lobe (MTL) activity. We carried out a preliminary test of this hypothesis by contrasting neural responses to stimuli eliciting either flashbacks or ordinary episodic memories of the same traumatic event in PTSD patients.

Previous research on the functional neuroanatomy of voluntary autobiographical memory has identified a core network of predominantly left-lateralized regions including the prefrontal cortex, MTL (in particular the hippocampus), and posterior cingulate (Svoboda, McKinnon, & Levine, 2006). Emotional memories, in contrast, are associated with bilateral activation and engage additional areas such as the amygdala and insula (Cabeza & St. Jacques, 2007; Svoboda et al., 2006). Emotion is thought to enhance recollection, and increased recollective qualities such as level of detail, personal significance, and emotionality have been found to be accompanied by increased MTL and hippocampal activity (Addis, Moscovitch, Crawley, & McAndrews, 2004; Svoboda & Levine, 2009; Wais, 2008).

Research on healthy participants therefore suggests that particularly detailed and emotional memories should be accompanied by high levels of activation in networks including hippocampal and parahippocampal regions. In contrast, the dual representation theory of PTSD distinguishes normal episodic memories, supported by flexible, contextualised representations, from flashbacks, supported by representations that are inflexible and lacking in context (Brewin, Gregory, Lipton, & Burgess, 2010). Although there is no formal definition of a flashback, they are commonly thought to involve an intense sensory and emotional re-experiencing of the traumatic event that exists on a continuum, ranging from complete loss of awareness of surroundings to a milder experience of reliving in the present (Brewin, 2011). This continuum view is part of the definition proposed for the upcoming fifth revision of the American Psychiatric Association’s Diagnostic and Statistical Manual (DSM-V) (Friedman, Resick, Bryant, & Brewin, 2011).

In the context of a basically intact episodic memory system, (visual) flashbacks are hypothesized by Brewin et al. (2010) to reflect the dominance during the traumatic event of activity in the dorsal visual stream, extending from posterior visual to superior parietal regions, that processes egocentric (i.e., own viewpoint), sensation-near representations of experience designed to facilitate action. These dorsal visual stream representations (sensation-based representations or S-reps) of the trauma are thought to be strongly associated with activity in motor cortex reflecting defensive actions as well as with activity in the insula and amygdala reflecting emotional and body state responses. Activity in the dorsal stream and its projections is hypothesized to take precedence over activity in the ventral visual stream, including inferior and middle temporal regions, that ordinarily enables scenes to be visualized allocentrically (i.e., from alternative viewpoints), and provides memories with their context (contextual representations or C-reps).

Most previous neuroimaging studies of PTSD have employed the script-driven imagery paradigm, in which participants simultaneously recall and imagine a traumatic event, a process which typically elicits additional involuntary trauma memories (Hopper, Frewen, Sack, Lanius, & van der Kolk, 2007). A case study was reported (Liberzon, Taylor, Fig, & Koeppe, 1996) in which a Vietnam veteran experienced a flashback during the perfusion phase of a SPECT scan. Relative to his own baseline, and to that of a PTSD sample who did not experience flashbacks, he exhibited decreases in blood flow to a wide range of cortical and subcortical regions. More recently subjective flashback intensity was correlated with rCBF in eleven PTSD patients (Osuch et al., 2001). They observed flashback-related increases in left inferior frontal cortex and bilateral insula, and flashback-related decreases in right medial temporal, right fusiform, and bilateral superior frontal cortices. Increased flashback reports in PTSD patients have also been found to be correlated with reduced brain volume in the left insula/parietal operculum and in the right inferior temporal gyrus (Kroes, Whalley, Rugg, & Brewin, 2011).

What are so far lacking are studies that directly compare flashbacks with ordinary episodic memories of the same traumatic event. In this study PTSD patients wrote a narrative account of their traumatic event, and identified which sections were and were not accompanied by flashbacks (Hellawell & Brewin, 2002, 2004). We subsequently presented them with words and phrases from these flashback and episodic memory sections mixed with stimuli from another narrative. In order to determine which patterns of observed activity were unique to PTSD, we employed control groups of depressed patients and healthy controls who had experienced a traumatic event. We predicted that recognition of own versus other stimuli should show common effects across groups in previously identified areas, reflecting an intact episodic memory system. We also predicted that the flashback-episodic trauma memory contrast would not show common across-group effects. Rather, the occurrence of flashbacks in PTSD patients would be distinguished by increased activity in dorsal stream and related areas such as motor cortex, insula, and amygdala, and by decreased ventral stream activity, for example in inferior and middle temporal areas.

## 2 Materials and methods

### 2.1 Participants

Participants were 39 right-handed individuals without a history of head injury, neurological disorders, or other major medical conditions, assessed using the Structured Clinical Interview for DSM-IV (First, Williams, & Spitzer, 1997). Ten patients met DSM-IV criteria for current PTSD (PTSD group). Fifteen participants had experienced traumatic events similar in magnitude to the PTSD group, but had not developed PTSD (trauma-exposed Control group). Fourteen patients meeting DSM-IV criteria for current major depression but not PTSD were also tested (depressed group). Patients in the PTSD and control groups had experienced a range of traumas, including involvement in the July 7th 2005 London bombings and other terrorist attacks (PTSD = 2; control = 7), road traffic accidents (PTSD = 1; control = 3), interpersonal violence (PTSD = 5; control = 3), the 2004 Asian tsunami (PTSD = 2; control = 1). Time since index trauma ranged from 2 months to 24 years, with a median of 2 years. Depressed patients had experienced a range of severe negative life events including some meeting PTSD Criterion A.

### 2.2 Overview of task

All participants visited the lab approximately a week prior to the scan and wrote a narrative account of their traumatic or most distressing event, starting from just before they knew something was wrong until the point where the event had resolved. Participants were prompted to include description of what they could see, hear, touch smell, taste, and feel at each stage of the incident, and also to describe their thoughts and emotions. For participants who reported multiple distressing experiences the event was chosen which bothered them most. At completion participants were invited to highlight any sections of the narrative during the writing of which they had experienced flashbacks. Flashbacks were defined for participants in the following way: “A type of memory that you experience as markedly different from those memories of an event that you can retrieve at will. The difference might be a marked sense of reliving of the traumatic experience(s). Some report complete reliving, whereas others report more momentary or partial reliving of perhaps just one aspect of the original experience. For some, flashback memories take them by surprise or swamp their mind. Finally, some report a sense of time-distortion and, for example, react to the flashback memory as though it was an event that was happening in the present”. Only PTSD patients identified flashbacks. Numbers of flashbacks were not counted separately.

Participants completed an autobiographical retrieval task, using stimuli culled from the written narratives, whilst brain activity was measured with fMRI. Participants were presented with items from their own narrative (Own), interspersed with items from another PTSD patient’s narrative thematically unrelated to their own (Other), and were asked to identify Own items. This type of cue-word task has previously been used in neuroimaging studies of autobiographical retrieval (Maguire, 2001; Maguire & Frith, 2003; Maguire & Mummery, 1999). For PTSD participants, Own items were additionally either associated with flashbacks they had of events (Flashback), or were simply normal episodic memories of the event (Episodic). Analyses were conducted to investigate successful autobiographical retrieval (Own > Other) and activity specific to flashbacks (OwnFlashback > OwnEpisodic).

### 2.3 Stimuli

Words (task 1) and phrases (task 2) were included to increase the number of stimuli that could be used without directly repeating items, and to generate additional opportunities for flashbacks to occur. Words and phrases (typically 2–8 words long) were chosen to be descriptive of key components of each narrative. Words were matched to ‘master lists’ (see later) on variables of word frequency and number of letters per word, and phrases was matched on the variables of number of words and letters per sentence (examples of master list items are given in supplementary materials). Two ‘master lists’ were generated to serve as control stimuli. One participant with PTSD had survived the July 2005 London bombings, the other had survived the December 2004 Asian tsunami. If a participant being tested had experienced one of these events then the alternative list was used. Certain words such as ‘blood’ or ‘helpless’ were common to many narratives, and words on the master list were substituted out on a case-by-case basis whenever overlap was identified.

### 2.4 Test procedure

Stimuli were presented via a mirror mounted on the head coil of the scanner, in direct view of the supine participant, at a distance of approximately 50 cm from the projection screen. Participants used an MR-compatible button-box in their right hand to respond and were instructed to respond as quickly and accurately as possible. For both tasks the presentation of an item was preceded by an asterisk (*) for 500 ms, followed by the item for (1000 ms in task 1, 1700 ms in task 2), followed by a fixation cross for (2000 ms in task 1, and 2000 ms in task 2). These sequences of events gave SOAs for tasks 1 and 2 of 3500 ms, and 4200 ms respectively. ‘Null events’ consisted of a fixation cross presented for an entire SOA.

In task 1 72 Own words and 72 Other words from one of the master lists were presented in the center of the screen in uppercase Arial font. 72 null-events were included whereby a fixation cross was presented for an entire SOA. Task 2 was identical but with a longer SOA to accommodate time for additional reading. Sixty Own phrases were presented along with 60 Other phrases in lowercase Arial font. After the scan PTSD participants were given lists of all the stimuli they had seen during the test and were required to identify whether or not each item had led to the experience of a flashback during the scan.

### 2.5 Data acquisition

MRI data were acquired on a 1.5 T whole body scanner (Magnetom Sonata, Siemens Medical, Erlangen, Germany) operated with an 8-channel phased array receive coil and the standard body transmit coil. The manufacturer’s standard automatic 3D-shim procedure was performed at the beginning of each experiment. The participants were scanned with a single-shot gradient-echo EPI sequence sensitive to the blood-oxygen level dependent (BOLD) effect using the following imaging parameters: 30 oblique transverse slices, slice thickness = 2.5 mm, gap between slices = 1.25 mm, repetition time TR = 3 s, flip angle *α* = 90^o^, echo time TE = 50 ms, readout bandwidth BW = 2298 Hz/pixel, bandwidth in PE direction BW_PE_ = 31.3 Hz/pixel, phase-encoding (PE) direction anterior–posterior, field of view FOV = 192 × 192 mm^2^, matrix size 64 × 64, fat suppression. BOLD sensitivity losses in the orbitofrontal cortex and the amygdala due to susceptibility artifacts were minimized by applying a *z*-shim gradient moment of −0.8 mT/m ms, a slice tilt of −30° and a positive PE gradient polarity (Weiskopf, Hutton, Josephs, & Deichmann, 2006). EPI magnitude images were reconstructed from the complex *k*-space raw data using a generalized reconstruction method based on the measured EPI *k*-space trajectory to minimize ghosting (Josephs, Deichmann, & Turner, 2000), combining the single coil images by sum of squares (Roemer, Edelstein, Hayes, Souza, & Mueller, 1990). EPI data acquisition was monitored on-line using a real-time reconstruction and quality assurance system (Weiskopf et al., 2007). Data were acquired during two separate sessions, with the first five volumes of each session discarded to allow for T1 equilibration effects. A magnetic (B0) field map image was collected before the first session and was used to unwarp the echo-planar images (Hutton et al., 2002; Hutton, Deichmann, Turner, & Andersson, 2004). Subjects were placed in a light head restraint within the scanner to limit head movement during acquisition. A 3D MDEFT T1-weighted structural image was also acquired following the functional acquisition for superimposing statistical maps over anatomy. Whole-brain structural scans were acquired using a Modified Driven Equilibrium Fourier Transform (MDEFT) sequence (Ugurbil et al., 1993) with optimized parameters as described in the literature (Deichmann, Schwarzbauer, & Turner, 2004). For each volunteer, 176 sagittal partitions were acquired with an image matrix of 256 × 224 (Read × Phase). Two-fold over-sampling was performed in read direction (head/foot direction) to prevent aliasing. The isotropic spatial resolution was 1 mm. Relevant imaging parameters were: TR/TE/TI = 12.24 ms/3.56 ms/530 ms.

### 2.6 Data analysis

fMRI data were processed and analyzed using the statistical software package SPM5 (Wellcome Trust Centre for Neuroimaging, London, UK. http://www.fil.ion.ucl.ac.uk/spm). The first 5 EPI volumes were discarded to allow for T1 equilibration. The remaining functional images from each subject were realigned using rigid-body transformation to correct for head movements to the mean functional image using 7th-degree B-spline interpolation, unwarped, and coregistered to the anatomical T1-weighted MR image using a normalized mutual information function. Next, structural images were segmented and normalized into a common stereotactic space (MNI 152 T1-template). Subsequently, the normalization parameters were applied to the functional images and these were resampled to 3 × 3 × 3-mm^3^ isotropic voxels 7th-degree B-spline interpolation. Finally, spatial smoothing was applied with an isotropic 3D Gaussian kernel of 8 mm full-width half-maximum.

Statistical analysis was performed in two stages of a mixed effects model. In the first stage the neural activity was modeled by a stick function (impulse event) at stimulus onset (task 1 – words) or an epoch with a duration of 1.5 s (task 2 – phrases). The ensuing BOLD response was modeled with a canonical haemodynamic response function (HRF) (Friston et al., 1998). An AR(1) model was used to estimate and correct for non-sphericity of the error covariance (Friston et al., 2002). For each voxel, the image time-series was high pass filtered to 1/128 Hz.

The resulting general linear model (GLM) was used to obtain parameter estimates representing the activity elicited by the events of interest. Four event types were defined, consisting of correct responses to ‘Own Flashback’ items, correct responses to ‘Own Episodic’ item, correct rejections to ‘Other Flashback’ items, and correct rejections to ‘Other Episodic’ items. For PTSD patients the Flashback/Episodic distinction was made by combining pre- and post-scan reports of which items were associated with flashbacks. Thus for this group any items which led to reports of a flashback, either during the narrative or while being scanned, were assigned to the ‘own flashback’ category. For control and depressed participants items were randomly assigned to ‘flashback’ and ‘episodic’ categories in order to allow identical data analysis procedures. Participants’ performance was high enough such that it was not possible to model misses. Also included for each session were six covariates to capture residual movement-related artifacts (three rigid-body translation, and three for rotation).

Contrasts for effects of interest were specified at the first level, and entered in to a full-factorial model at the second level. Run 1 (words) and run 2 (phrases) were included as separate sessions within the second-level model, but to maximize power, and because we had no a-priori hypotheses requiring separate analyses, all results presented here are reported for the combination of words and sentences tasks. Based on an analysis of demographic data covariates of no interest ‘length of time since trauma’, and ‘BDI’ were included in the model.

All effects were thresholded at *p* < 0.001 with an extent of *k* > 5 unless otherwise specified. Between-group effects were generated by interrogating the model discussed above. Common effects (effects common to the PTSD, control and depressed groups) were obtained by an inclusive mask of activity in each of the three groups, each thresholded at *p* < 0.025 to give a conjoint threshold, via Fisher’s procedure, of *p* < 0.001 (conjunction null) (Nichols, Brett, Andersson, Wager, & Poline, 2005).

## 3 Results

### 3.1 Demographic and behavioral data

Patient demographics and scores on clinical measures are given in Table 1. Groups did not differ according to age or the age at which they left full-time education, but post hoc tests indicated that the depressed group had experienced their index event significantly longer ago than the controls. BDI and BAI scores for the PTSD and depressed groups did not differ, but both were significantly higher than the controls. Participants in the PTSD group scored significantly higher than controls on the PDS. Nine of the depressed group, 3 of the PTSD group, and none of the control group were taking antidepressant medication.

Memory performance is given in Table 2. Pr provides an unbiased estimate of accuracy in the response to old and new items, where higher values correspond to greater accuracy. Br is an index of response bias, the tendency to respond “old” or “new” regardless of accuracy (Snodgrass & Corwin, 1988).

There was a main effect of task on Pr, *F*(1, 36) = 283.42, *p* < 0.001, but no main effect of group or task by group interaction. Pr was significantly higher for phrases (mean 0.79, SD 0.09) than for words (mean 0.55, SD 0.12), *t*(38) = 16.77, *p* < 0.001. There was also a main effect of task on Br, *F*(1, 36) = 17.02, *p* < 0.001, but no main effect of group or task by group interaction. Br was significantly higher for words (mean 0.47, SD 0.14) than for phrases (mean 0.32, SD 0.22), *t*(38) = 4.366, *p* < 0.001. Response bias for the words task was neutral but for the sentences task was < 0.5, indicating a propensity for participants to respond “new”. This pattern is driven by the very low rate of false alarms: participants were very accurate in correctly rejecting phrases from another individual’s narrative but were more cautious identifying phrases as being from their own narrative.

Patients in the PTSD group reported flashbacks to an average of 50.4 out of a total of 264 individual stimuli. Of these, an average of 31.6 were to words or phrases that had been reported as eliciting a flashback during the narrative task, and an average of 15.5 were to words or phrases that had not been reported as eliciting a flashback during the narrative task. The remaining 3.3 flashbacks were to words or phrases from the control (Other) narrative. As the focus of interest was the experience of a flashback, all these items were included in the analyses.

### 3.2 fMRI data

**Fig. 1:**
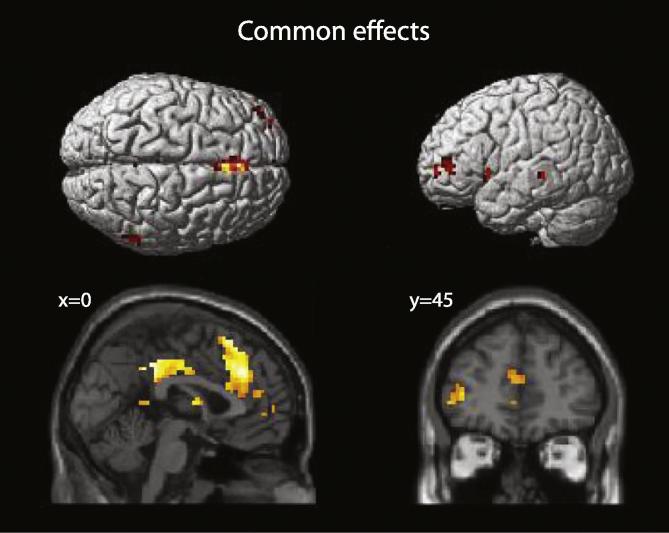
Effects common to all three groups for the Own > Other contrast. Results thresholded at *p* < 0.001, *k* > 5.

Analyses were first directed at finding activity common to all three groups during the successful retrieval of autobiographical memories. Subsequent analyses aimed to delineate between-group differences. Unless otherwise specified all contrasts reported here include the variables ‘BDI’ and ‘time since trauma’ as covariates of no interest. Effects common to all three groups for the Own > Other contrast (i.e. hits > correct rejections) are given in Table 3 and Fig. 1. There was significant activity in the left middle, superior medial, and superior frontal cortices. Bilateral posterior cingulate, right caudate, left insula, left retrosplenial cortex, and left middle temporal cortex also demonstrated activation. Minimal between-group effects were observed for the Own > Other contrast. Relative to the depressed and control groups, the PTSD group demonstrated increased activity in a 5-voxel region of the right mid-cingulate cortex (*x* = 9, *y* = 9, *z* = 45), and decreased activity in an 8-voxel region of the right inferior frontal gyrus (*x* = 45, *y* = 33, *z* = −3).

**Fig. 2:**
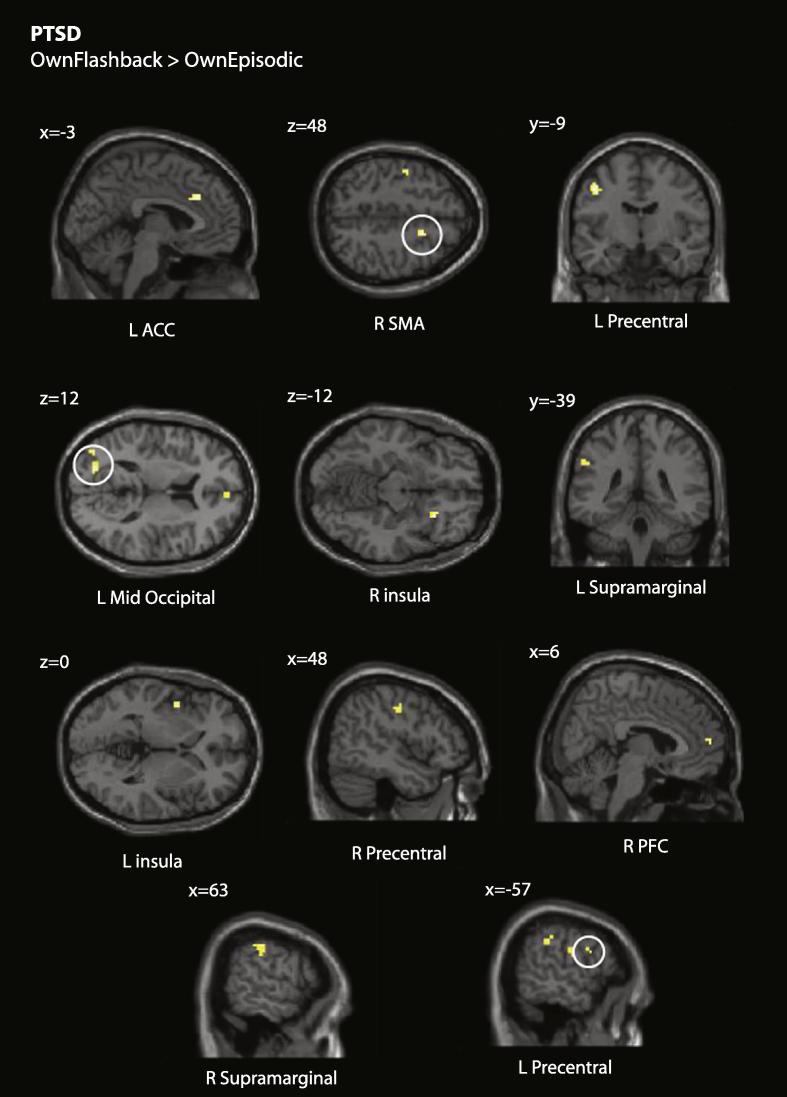
Flashback-specific activity in the PTSD group. Results thresholded at *p* < 0.001, *k* > 5.

We next examined activity associated with the OwnFlashback > OwnEpisodic contrast. There were no effects common to all three groups. Table 4 and Fig. 2 report activations associated with flashbacks versus episodic memories in the PTSD group only. Flashback-associated increases in activations were evident in left anterior cingulate (ACC) and middle occipital cortices, right supplementary motor area (SMA) and medial prefrontal cortex, and bilaterally in precentral areas, inferior parietal (supramarginal) cortices, and the insula.

**Fig. 3:**
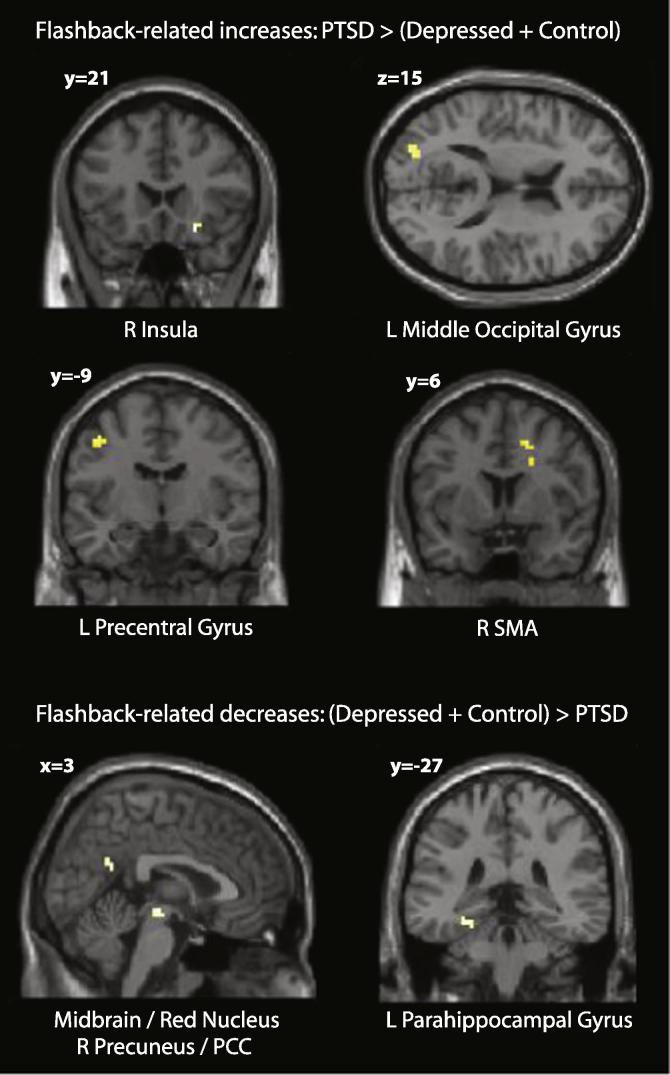
Flashback-specific between-groups differences. Results thresholded at *p* < 0.001, *k* > 5.

**Fig. 4:**
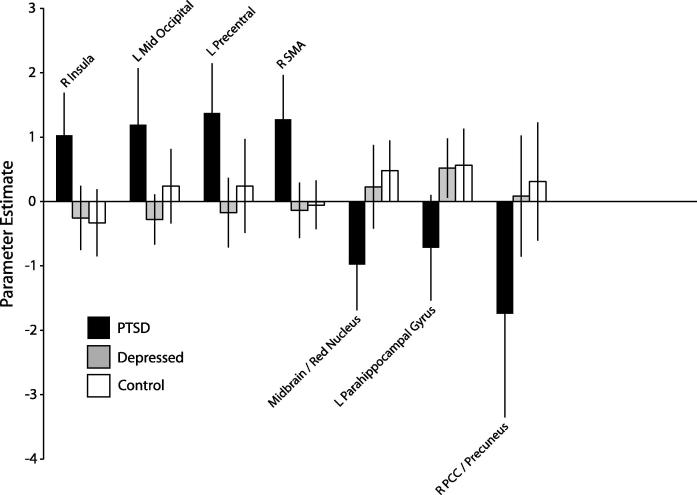
Parameter estimates extracted from the peak voxel of each of the key regions identified in the between-subjects flashbacks analysis. Error bars show 95% confidence interval.

We further investigated regions demonstrating flashback-associated increases or decreases in the PTSD group relative to the control and depressed groups. These are given in Table 5 and Fig. 3. Parameter estimates for each group are given in Fig. 4. There was a substantial overlap with the preceding analysis, PTSD patients showing significantly greater flashback-specific activity in regions of the right SMA, left middle occipital gyrus and precentral cortex, and right insula. In addition PTSD patients showed reduced flashback-specific activity in the midbrain/red nucleus, left fusiform/parahippocampal gyrus, and right precuneus/PCC.

## 4 Discussion

Behavioral results indicated that participants in all groups were able to identify their own autobiographical events with a high degree of accuracy. This contrasts with evidence for an overall deficit in PTSD in learning neutral material (Brewin, Kleiner, Vasterling, & Field, 2007). Separate analyses not reported here showed that the occurrence of flashbacks was in general associated with greater accuracy, but on those rare occasions when they occurred to stimuli from another person’s narrative those stimuli were likely to be falsely recognized as belonging to their own narrative (Brewin, Huntley, & Whalley, 2012).

Neural effects common to all three groups during the successful retrieval of autobiographical memories were observed in several prefrontal areas as well as the posterior cingulate, precuneus, and retrosplenial cortex, regions identified as primary areas of the autobiographical memory system (Gilboa, 2004; Svoboda et al., 2006). As in previous studies of emotional memory (Cabeza & St. Jacques, 2007; Svoboda et al., 2006), insula activity was also common to all groups. Between-group differences for the Own > Other contrast were much more limited. Relative to the other groups PTSD patients showed increased activity in the right mid-cingulate cortex, and reduced activity in the right inferior frontal cortex. This limited pattern of differential activity is consistent with previous work (Gilboa et al., 2004; Whalley, Rugg, Smith, Dolan, & Brewin, 2009) in suggesting a substantially intact neural system supporting episodic retrieval.

As predicted, given the absence of flashbacks reported by depressed patients and controls, there were no effects common to all three groups for the OwnFlashback > OwnEpisodic contrast. Relative to the depressed and control groups, PTSD participants exhibited flashback-specific increases in the right insula, left mid-occipital cortex, left precentral cortex, and right SMA, and decreases in the midbrain (red nucleus), the left parahippocampal gyrus, and the right precuneus. These results were broadly consistent with the hypotheses: As well as increases in the insula and mid-occipital areas, corresponding to the affective and visual components of S-reps predicted by dual representation theory, changes in activation were observed in a number of recognized pathways emerging from the dorsal visual stream.

The dorsal visual stream has two parallel projections to dorsal and ventral regions of premotor cortex (Kravitz, Saleem, Baker, & Mishkin, 2011), which are believed to mediate eye movements, reaching, and grasping, among other forms of visually guided action that may be required for defensive purposes. These pathways could account for the observed activations in SMA and precentral areas, which are also consistent with previous studies of PTSD (Gilboa et al., 2004). A connectivity analysis (Vogt, Vogt, & Laureys, 2006) has also linked body orientation in space to a network that includes the SMA and precentral gyrus.

The results were also consistent with the prediction that intense flashback-type memories would be associated with a decrease in activity in areas such as middle and inferior temporal cortex, associated with allocentric spatial representations. In addition to decreased activity in the parahippocampus, a ventral visual stream region involved in allocentric scene information (Burgess, Becker, King, & O’Keefe, 2001), we found reduced activation in an area including the posterior cingulate cortex (PCC) and precuneus. The PCC is involved in the conversion of egocentric to allocentric representations, essentially contextualising a sensory representation.

The precuneus is a complex area subserving a number of different functions. Whereas the anterior precuneus is mainly connected to sensorimotor areas, also including a projection to the insula, the central precuneus is viewed as a cognitive/association area (Margulies et al., 2009). It has been argued that the precuneus is part of an important network subserving consciousness, and that during aversive sensations there may be efforts to terminate self-reflection, resulting in decreased processing in the precuneus (Vogt & Laureys, 2005). Flashbacks are certainly experienced as aversive by PTSD patients, as indicated by the fact that avoidance of involuntary trauma memories forms part of the diagnostic criteria, and our results are consistent with this process. Alternatively, reduced precuneus activation may be connected with the involuntary nature of memory retrieval (Hall, Gjedde, & Kupers, 2008).

The red nucleus has been shown to be part of an extended functional network that includes associative prefrontal, parietal, occipital, temporal, and limbic areas, but not sensorimotor cortex (Nioche, Cabanis, & Habas, 2009). Nioche et al. argue that its widespread connectivity points towards a cognitive role, possibly related to salience detection or executive control. An activity decrease here during overwhelming flashback experiences could indicate a disruption to normal executive processes, or might reflect disrupted patterns of input and output to and from other flashback-affected areas.

Analyses of flashbacks within the PTSD group alone found additional evidence for greater activation of motor cortex and other closely connected regions involved in response selection such as dorsal ACC (dACC). This is of particular interest as dorsal ACC is thought to modulate fear expression in humans (Milad et al., 2007). There is evidence for hyperresponsivity in some areas of dACC being a familial risk factor for PTSD (Shin et al., 2011).

Analyses of flashbacks in the PTSD group revealed bilateral activation in the supramarginal gyrus. This forms part of the temporo-parietal junction (TPJ), an area involved in self-location and first-person perspective (Ionta et al., 2011). Consistent with predictions from dual representation theory, right-lateralized activation in the same area we identified in this study may be associated with the ability to represent or act on objects from an egocentric perspective (Maguire et al., 1998).

Within the memory literature this inferior parietal area is also hypothesized to be involved in the capture of attention bottom-up by behaviorally-relevant stimuli (Cabeza, Ciaramelli, Olson, & Moscovitch, 2008) Cabeza et al.’s Attention to Memory model specifically hypothesized that not just external stimuli but also internal stimuli such as salient memories could activate this area, and specifically suggested activation as a result of involuntary remembering. Subsequent analyses have shown greater connectivity between the supramarginal gyrus and occipital areas when a salient perceptual stimulus is detected, but greater connectivity with the MTL when a salient memory is detected (Cabeza et al., 2011). Flashbacks are perhaps the most extreme example of attentionally-salient involuntary remembering, including very marked perceptual features, and so provide an excellent test of this hypothesis. Together with recent work on the consequences of failed attempts at memory suppression (Levy, Huddleston, & Anderson, 2011), our findings are among the first to confirm Cabeza et al.’s prediction. Ventral parietal activations tend to be left-sided when recalling episodic memories and right-lateralized for perceptual stimuli but many perceptual studies find bilateral activations consistent with our data.

The neural activity distinguishing the OwnFlashback > OwnEpisodic contrast in PTSD patients has some striking similarities with other areas of the memory literature, for example with the neural correlates of recollection and familiarity (Dörfel, Werner, Schaefer, von Kummer, & Karl, 2009). These authors identified precuneus activity that was common to recollection and familiarity, but found that it was lower for familiarity than for recollection. They also reported that hippocampal and parahippocampal activation was associated with recollection but not familiarity, whereas supplementary motor area activity was more strongly associated with familiarity than with recollection. This suggests there may be some connection between the experience of a flashback and a familiarity judgement.

Memory studies using healthy controls and stimuli of low to average personal significance have found that recollection of the learning event, accompanied by its contextual detail, is normally associated with greater MTL activity. In addition the posterior midline region, medial prefrontal cortex, and posterior parietal cortex contribute to a separate network related to decisions concerning retrieval success (Henson, Hornberger, & Rugg, 2005; Huijbers, Pennartz, & Daselaar, 2010; Wagner, Shannon, Kahn, & Buckner, 2005). Although it is unclear whether activity in these regions reflects episodic long-term memory or simply retrieval confidence, it has been suggested that they could support judgments that a retrieval cue was familiar in the absence of actual recollection. For example, the mnemonic accumulator hypothesis (Wagner et al., 2005) proposed that areas in posterior parietal cortex integrate and accumulate available mnemonic evidence relevant to decisions about whether a cue is old versus new.

It is natural to assume that very intensely experienced memories such as flashbacks would exemplify a particularly strong form of recollection, particularly since in the average laboratory study familiarity judgments are usually made in the absence of any remembered detail. Recent evidence indicates, however, that in some instances familiarity judgements may also be very strong (Ingram, Mickes, & Wixted, 2012). Posttraumatic stress disorder is characterized by images that, although lacking in spatial and temporal context, are richly detailed and persistent, and accompanied by autonomic and body state elements. Such images could similarly have the potential to provide strong mnemonic evidence that a traumatic event has occurred. It is possible that when such images are retrieved they are treated like the cues presented in standard recognition studies, only eliciting a decision about their familiarity rather than any further elaboration and recollection.

As already noted, a cardinal symptom of PTSD is avoidance of thoughts and memories concerning the trauma, which suggests that elaboration of a very negative memory would be aversive. This tendency toward avoidance, which may be illustrated in PTSD by the finding of a negative influence of prefrontal cortex on visual areas such as the precuneus in response to trauma reminders (Gilboa et al., 2004), is specifically counteracted in trauma-focused cognitive–behavior therapy (Ehlers & Clark, 2000).

We propose therefore that successful identification of flashbacks is based on a strong feeling of familiarity. Full recollection is not required because the detailed sensory dorsal-stream representations enable the individual to identify the images rapidly and with great confidence without further memory search. It should be noted, however, that the stimuli in our study were presented more briefly than in the average autobiographical memory investigation, and this provides a possible alternative explanation for the failure to observe classic markers of recollection in the neural response to flashbacks.

This preliminary study was subject to a number of significant limitations. Participants had experienced a heterogeneous mix of trauma exposure, there was a wide range of time elapsed since the trauma, and they included a mix of medicated and unmedicated patients. Although it has been argued that neuroimaging studies should include medicated and unmedicated patients in order to reflect the general PTSD population (Lanius et al., 2010), not enough is known about potential interactions of drug and task. There were insufficient numbers in this study to examine drug effects statistically. There was also significant comorbidity in our patient sample, with high levels of depressive symptoms, although our use of a separate depressed group as well as the inclusion of depression as a covariate attempted to control for this.

Not all PTSD participants had flashbacks in the scanner. Limiting our analyses purely to items which were associated with in-scanner flashbacks would have been desirable but there were insufficient data. Our ‘flashback’ category was therefore an amalgam of items which led to flashbacks during the writing of the narrative *and* items which caused a flashback in the scanner (generally an Own but occasionally an Other item). Confirmation of our findings must await replication with a larger sample that can offer more statistical power. Finally, we did not gather data about how participants were responding to flashbacks. There is a considerable literature examining attempts to suppress or reappraise emotional memories which could be applied to this question.

## 5 Conclusions

Our results confirm that the episodic memory system in PTSD is likely intact, and provide some preliminary evidence that flashbacks may be associated with increases in activity in areas associated with the dorsal visual stream, coupled with decreases in ventral stream activity. Our data also support previous indications that PTSD patients can discriminate the occurrence of flashbacks during written narratives (Hellawell & Brewin, 2002, 2004). Consistent with the fact that the parts of narratives that involve flashbacks contain more motion words (Hellawell & Brewin, 2004), the increased activations in numerous areas of motor cortex suggest that flashbacks are a form of memory that facilitates action on the environment (such as fight or flight).

The data may also have a bearing on the controversy concerning dual-process models of recognition memory. Although comparisons of recollection and familiarity are often confounded with strong and weak memories respectively (Squire, Wixted, & Clark, 2007; Wixted, 2007), these two processes may nevertheless have separate neural substrates (Dörfel et al., 2009; Rugg & Yonelinas, 2003; Yonelinas, Otten, Shaw, & Rugg, 2005). Our findings suggest that even intense autobiographical memories, experienced with extreme clarity and vividness, may sometimes demonstrate a neural signature that more closely resembles familiarity than recollection.

## 6 Funding

This work was supported by Wellcome Trust Grant No. 074145.

## Figures and Tables

**Table 1:** Means (standard deviations) of demographic and clinical data by group.

	PTSD	Depressed	Control	F(2, 36)
Age	38.6 (7.66)	34.5 (7.47)	33.9 (8.21)	1.25
Age left education	21.0 (5.01)	22.07 (3.29)	22.53 (3.30)	0.48
Time since index trauma (months)	60.8ab (85.41)	121.84a (105.39)	35.66b (23.80)	3.65⁎
Beck depression inventory	27.10a (10.26)	29.21a (8.06)	6.26b (5.50)	28.77⁎⁎
Beck anxiety inventory	23.33a (12.18)	18.5a (10.89)	6.20b (6.67)	10.41⁎⁎
				*t*(23)
Posttraumatic diagnostic scale	33.5 (8.33)	–	8.33 (5.31)	8.56⁎⁎

Different subscripts indicate significant differences (*p* < .05).

⁎ *p* < .05.

⁎⁎ *p* < .001.

**Table 2:** Memory accuracy (Pr and Br).

Memory accuracy	PTSD	Depressed	Control
*Words*			
Pr	0.514 (0.14)	0.590 (0.12)	0.525 (0.09)
Br	0.544 (0.13)	0.432 (0.16)	0.473 (0.12)

*Phrases*			
Pr	0.793 (0.12)	0.809 (0.09)	0.777 (0.09)
Br	0.422 (0.22)	0.261 (0.22)	0.324 (0.22)

**Table 3:** Common effects for Own > Other contrast. Inclusive mask of PTSD, Depressed, and Control results. Each thresholded at *p* = 0.025 to produce combined threshold of *p* = 0.001 (via Fisher’s procedure). BA indicates Brodmann area; MNI, Montreal Neurological Institute.

Region	BA	Cluster size	MNI coordinates (*x* *y* *z*)
L Superior medial gyrus	9	281	0 33 33
L PCC/Precuneus	23	11	−3 −45 36
L/R Posterior cingulate	23/31	82	0 −39 39
L Superior frontal gyrus	10	10	−33 57 0
R Caudate nucleus		56	9 9 3
L Insula	13	8	−45 12 0
R Supramarginal gyrus	40	10	60 −48 36
L Middle Frontal gyrus	10/46	26	−39 45 6
L Retrosplenial cortex	26/29/30	6	−3 −45 9
L Middle Temporal gyrus	21	8	−54 −33 −3

**Table 4:** Regions demonstrating increased activation for the OwnFlashback > OwnEpisodic contrast in the PTSD group only. Thresholded at *p* < 0.001, *k* > 5. No covariates were included in this analysis. BA indicates Brodmann area; MNI, Montreal Neurological Institute.

Region	BA	Cluster size	MNI coordinates (*x* *y* *z*)	Z-score
L Anterior cingulate	24	43	−3 27 30	4.17
R SMA	6	20	18 9 48	4.08
L Precentral	6	58	−45 −9 42	3.93
L Middle occipital		35	−21 −78 12	3.79
R Insula	13/14	7	27 21 −12	3.72
L Supramarginal	40	17	−54 −39 33	3.58
L Insula	13/14	8	−42 3 0	3.56
R Precentral		12	48 −15 42	3.53
R Superior medial PFC	10	5	6 57 12	3.49
R Supramarginal		21	63 −27 42	3.39
L Precentral		5	−57 3 27	3.29

**Table 5:** Regions demonstrating increased or decreased flashback-associated activity in the PTSD group relative to the control and depressed groups. Thresholded at *p* < 0.001, *k* > 5. These results include ‘length of time since trauma’ and ‘BDI’ as covariates of no interest. BA indicates Brodmann area; MNI, Montreal Neurological Institute.

Region	BA	Cluster size	MNI coordinates (*x* *y* *z*)	Z-score
*PTSD > (Depressed + Control)*				
R Insula	13/14	6	24 21 −15	4.26
L Middle occipital gyrus		18	−24 −81 15	3.85
L Precentral gyrus	6	11	−42 −9 45	3.67
R supplementary motor area (SMA)	6	5	21 6 42	3.36

*(Depressed + Control) > PTSD*				
Midbrain – red nucleus		8	3 −21 −9	3.63
L Parahippocampal gyrus	36/37	6	−27 −36 −15	3.43
R Precuneus/PCC	31	11	3 −57 24	3.37

## References

[b0005] Addis D.R., Moscovitch M., Crawley A.P., McAndrews M.P. (2004). Recollective qualities modulate hippocampal activation during autobiographical memory retrieval. Hippocampus.

[b0010] Brewin C.R. (2001). A cognitive neuroscience account of posttraumatic stress disorder and its treatment. Behaviour Research and Therapy.

[b0015] Brewin C.R. (2011). The nature and significance of memory disturbance in posttraumatic stress disorder. Annual Review of Clinical Psychology.

[b0020] Brewin C.R., Dalgleish T., Joseph S. (1996). A dual representation theory of posttraumatic stress disorder. Psychological Review.

[b0025] Brewin C.R., Gregory J.D., Lipton M., Burgess N. (2010). Intrusive images in psychological disorders: Characteristics, neural mechanisms, and treatment implications. Psychological Review.

[b0030] Brewin C.R., Huntley Z., Whalley M.G. (2012). Source memory errors associated with reports of posttraumatic flashbacks: A proof of concept study. Cognition.

[b0035] Brewin C.R., Kleiner J.S., Vasterling J.J., Field A.P. (2007). Memory for emotionally neutral information in posttraumatic stress disorder: A meta-analytic investigation. Journal of Abnormal Psychology.

[b0040] Burgess N., Becker S., King J.A., O’Keefe J. (2001). Memory for events and their spatial context: Models and experiments. Philosophical Transactions of the Royal Society of London. Series B, Biological Sciences.

[b0045] Cabeza R., Ciaramelli E., Olson I.R., Moscovitch M. (2008). The parietal cortex and episodic memory: An attentional account. Nature Reviews Neuroscience.

[b0050] Cabeza R., Mazuz Y.S., Stokes J., Kragel J.E., Woldorff M.G., Ciaramelli E. (2011). Overlapping parietal activity in memory and perception: Evidence for the Attention to Memory model. Journal of Cognitive Neuroscience.

[b0055] Cabeza R., St. Jacques P. (2007). Functional neuroimaging of autobiographical memory. Trends in Cognitive Sciences.

[b0060] Deichmann R., Schwarzbauer C., Turner R. (2004). Optimisation of the 3D MDEFT sequence for anatomical brain imaging: Technical implications at 1.5 and 3 T. Neuroimage.

[b0065] Dörfel D., Werner A., Schaefer M., von Kummer R., Karl A. (2009). Distinct brain networks in recognition memory share a defined region in the precuneus. European Journal of Neuroscience.

[b0070] Ehlers A., Clark D.M. (2000). A cognitive model of posttraumatic stress disorder. Behaviour Research and Therapy.

[b0075] First M.B., Williams J.B., Spitzer R.L. (1997). Structured clinical interview for DSM-IV axis I disorders (SCID-I).

[b0080] Friedman M.J., Resick P.A., Bryant R.A., Brewin C.R. (2011). Considering PTSD for DSM-5. Depression and Anxiety.

[b0085] Friston K.J., Fletcher P., Josephs O., Holmes A., Rugg M.D., Turner R. (1998). Event-related fMRI: Characterizing differential responses. Neuroimage.

[b0090] Friston K.J., Penny W., Phillips C., Kiebel S., Hinton G., Ashburner J. (2002). Classical and Bayesian inference in neuroimaging: Theory. Neuroimage.

[b0095] Gilboa A. (2004). Autobiographical and episodic memory – one and the same? Evidence from prefrontal activation in neuroimaging studies. Neuropsychologia.

[b0100] Gilboa A., Shalev A.Y., Laor L., Lester H., Louzoun Y., Chisin R. (2004). Functional connectivity of the prefrontal cortex and the amygdala in posttraumatic stress disorder. Biological Psychiatry.

[b0105] Hall N.M., Gjedde A., Kupers R. (2008). Neural mechanisms of voluntary and involuntary recall: A PET study. Behavioural Brain Research.

[b0110] Hellawell S.J., Brewin C.R. (2002). A comparison of flashbacks and ordinary autobiographical memories of trauma: Cognitive resources and behavioural observations. Behaviour Research and Therapy.

[b0115] Hellawell S.J., Brewin C.R. (2004). A comparison of flashbacks and ordinary autobiographical memories of trauma: Content and language. Behaviour Research and Therapy.

[b0120] Henson R.N.A., Hornberger M., Rugg M.D. (2005). Further dissociating the processes involved in recognition memory: An fMRI study. Journal of Cognitive Neuroscience.

[b0125] Hopper J.W., Frewen P.A., Sack M., Lanius R.A., van der Kolk B.A. (2007). The responses to script-driven imagery scale (RSDI): Assessment of state posttraumatic symptoms for psychobiological and treatment research. Journal of Psychopathological and Behavioral Assessment.

[b0130] Huijbers W., Pennartz C.M.A., Daselaar S.M. (2010). Dissociating the “retrieval success” regions of the brain: Effects of retrieval delay. Neuropsychologia.

[b0135] Hutton C., Bork A., Josephs O., Deichmann R., Ashburner J., Turner R. (2002). Image distortion correction in fMRI: A quantitative evaluation. Neuroimage.

[b0140] Hutton, C., Deichmann, R., Turner, R., Andersson, J. L. R. (2004). Combined correction for geometric distortion and its interaction with head motion in fMRI. In *Paper presented at the Proceedings of ISMRM 12*, Kyoto, Japan.

[b0145] Ingram K.M., Mickes L., Wixted J.T. (2012). Recollection can be weak and familiarity can be strong. Journal of Experimental Psychology. Learning, Memory, and Cognition.

[b0150] Ionta S., Heydrich L., Lenggenhager B., Mouthon M., Fornari E., Chapuis D. (2011). Multisensory mechanisms in temporo-parietal cortex support self-location and first-person perspective. Neuron.

[b0155] Josephs O., Deichmann R., Turner R. (2000). Trajectory measurement and generalised reconstruction in rectilinear EPI. Neuroimage.

[b0160] Kravitz D.J., Saleem K.S., Baker C.I., Mishkin M. (2011). A new neural framework for visuospatial processing. Nature Reviews Neuroscience.

[b0165] Kroes M.C.W., Whalley M.G., Rugg M.D., Brewin C.R. (2011). Association of flashbacks and structural brain abnormalities in posttraumatic stress disorder. European Psychiatry.

[b0170] Lanius R.A., Brewin C.R., Bremner J.D., Daniels J.K., Friedman M.J., Liberzon I. (2010). Does neuroimaging research examining the pathophysiology of posttraumatic stress disorder require medication-free patients?. Journal of Psychiatry and Neuroscience.

[b0175] Levy, B. J., Huddleston, E., Anderson, M. C., (20110. Failed memory suppression reflects attentional capture within memory. In *Paper presented at the 5th international conference on memory*, York, UK.

[b0180] Liberzon I., Taylor S.F., Fig L.M., Koeppe R.A. (1996). Alteration of corticothalamic perfusion ratios during a PTSD flashback. Depression and Anxiety.

[b0185] Maguire E.A. (2001). Neuroimaging studies of autobiographical event memory. Philosophical Transactions of the Royal Society of London. Series B, Biological Sciences.

[b0190] Maguire E.A., Burgess N., Donnett J.G., Frackowiak R.S.J., Frith C.D., O’Keefe J. (1998). Knowing where and getting there: A human navigation network. Science.

[b0195] Maguire E.A., Frith C.D. (2003). Lateral asymmetry in the hippocampal response to the remoteness of autobiographical memories. Journal of Neuroscience.

[b0200] Maguire E.A., Mummery C.J. (1999). Differential modulation of a common memory retrieval network revealed by positron emission tomography. Hippocampus.

[b0205] Margulies D.S., Vincent J.L., Kelly C., Lohmann G., Uddin L.Q., Biswal B.B. (2009). Precuneus shares intrinsic functional architecture in humans and monkeys. Proceedings of the National Academy of Sciences of the United States.

[b0210] Milad M.R., Quirk G.J., Pitman R.K., Orr S.P., Fischl B., Rauch S.L. (2007). A role for the human dorsal anterior Cingulate cortex in fear expression. Biological Psychiatry.

[b0215] Nichols T., Brett M., Andersson J., Wager T., Poline J.B. (2005). Valid conjunction inference with the minimum statistic. Neuroimage.

[b0220] Nioche C., Cabanis E.A., Habas C. (2009). Functional connectivity of the human red nucleus in the brain resting state at 3T. American Journal of Neuroradiology.

[b0225] Osuch E.A., Benson B., Geraci M., Podell D., Herscovitch P., McCann U.D. (2001). Regional cerebral blood flow correlated with flashback intensity in patients with posttraumatic stress disorder. Biological Psychiatry.

[b0230] Roemer P.B., Edelstein W.A., Hayes C.E., Souza S.P., Mueller O.M. (1990). The NMR phased array. Magnetic Resonance in Medicine.

[b0235] Rugg M.D., Yonelinas A.P. (2003). Human recognition memory: A cognitive neuroscience perspective. Trends in Cognitive Sciences.

[b0240] Shin L.M., Bush G., Milad M.R., Lasko N.B., Brohawn K.H., Hughes K. (2011). Exaggerated activation of dorsal anterior cingulate cortex during cognitive interference. A monozygotic twin study of posttraumatic stress disorder. American Journal of Psychiatry.

[b0245] Snodgrass J.G., Corwin J. (1988). Pragmatics of measuring recognition memory: Applications to dementia and amnesia. Journal of Experimental Psychology: General.

[b0250] Squire L.R., Wixted J.T., Clark R.E. (2007). Recognition memory and the medial temporal lobe: A new perspective. Nature Reviews Neuroscience.

[b0255] Svoboda E., Levine B. (2009). The effects of rehearsal on the functional neuroanatomy of episodic autobiographical and semantic remembering: A functional magnetic resonance imaging study. Journal of Neuroscience.

[b0260] Svoboda E., McKinnon M.C., Levine B. (2006). The functional neuroanatomy of autobiographical memory: A meta-analysis. Neuropsychologia.

[b0320] Ugurbil K., Garwood M., Ellermann J., Hendrich K., Hinke R., Hu X. (1993). Imaging at high magnetic fields: Initial experiences at 4T. Magnetic Resonance Quarterly.

[b0265] van der Kolk B.A., van der Kolk B.A., McFarlane A.C., Weisaeth L. (1996). Trauma and memory. Traumatic stress.

[b0270] Vogt B.A., Laureys S. (2005). Posterior cingulate, precuneal and retrosplenial cortices: Cytology and components of the neural network correlates of consciousness. Progress in Brain Research.

[b0275] Vogt B.A., Vogt L., Laureys S. (2006). Cytology and functionally correlated circuits of human posterior cingulate areas. Neuroimage.

[b0280] Wagner A.D., Shannon B.J., Kahn I., Buckner R.L. (2005). Parietal lobe contributions to episodic memory retrieval. Trends in Cognitive Sciences.

[b0285] Wais P.E. (2008). FMRI signals associated with memory strength in the medial temporal lobes: A meta-analysis. Neuropsychologia.

[b0290] Weiskopf N., Hutton C., Josephs O., Deichmann R. (2006). Optimal EPI parameters for reduction of susceptibility-induced BOLD sensitivity losses: A whole-brain analysis at 3 T and 1.5 T. Neuroimage.

[b0295] Weiskopf N., Sitaram R., Josephs O., Veit R., Scharnowski F., Goebel R. (2007). Real-time functional magnetic resonance imaging: Methods and applications. Magnetic Resonance Imaging.

[b0300] Whalley M.G., Farmer E., Brewin C.R. (2007). Pain flashbacks following the July 7th 2005 London bombings. Pain.

[b0305] Whalley M.G., Rugg M.D., Smith A.P.R., Dolan R.J., Brewin C.R. (2009). Incidental retrieval of emotional contexts in post-traumatic stress disorder and depression: An fMRI study. Brain and Cognition.

[b0310] Wixted J.T. (2007). Dual-process theory and signal-detection theory of recognition memory. Psychological Review.

[b0315] Yonelinas A.P., Otten L.J., Shaw K.N., Rugg M.D. (2005). Separating the brain regions involved in recollection and familiarity in recognition memory. Journal of Neuroscience.

